# Data on race, inequality, and social capital in the U.S. counties

**DOI:** 10.1016/j.dib.2021.106717

**Published:** 2021-01-07

**Authors:** Dongkyu Kim, Mi-son Kim, Natasha Altema McNeely

**Affiliations:** Department of Political Science, University of Texas Rio Grande Valley, 211 ELABN, 1201W. University Dr. Edinburg, TX 78539, United States

**Keywords:** Racial diversity, Income inequality, Social capital, U.S. counties

## Abstract

This article presents data on social capital at the United States’ county-level. Following Rupasingha et al. (2006), the social capital index captures the common factor among density measures of 10 different types of associations, voter turnout rates, U.S. decennial census participation rates, and the number of non-profit organizations. Based on Knack (2003), we create associational densities measures as a proxy for both bridging and bonding social capital. Including data on income inequality, racial diversity, minority group size, average household income, educational attainment, the ratio of a family household, the size of migration population, and female labor market participation rates, the data covers 3,104 U.S. counties for both 2009 and 2014. This paper includes descriptive statistics and figures. This data article is associated with the article “Race, Inequality, and Social Capital in the U.S. Counties.”

## Specifications Table

**Table:** 

Subject	Sociology and Political Science
Specific subject area	Racial diversity, income inequality, social capital in the U.S. counties
Type of data	Comma-separated values, tables, figures
How data were acquired	The original data are from the websites of the Northeast Regional Center for Rural Development at Penn State University, the American Community Survey of the U.S. Census Bureau, and the U.S. Department of Agriculture.
Data format	Comma Separated values & Analysed
Parameters for data collection	All U.S. counties for both 2009 and 2014
Description of data collection	The Northeast Regional Center for Rural Development at Penn State University provides raw data on the social capital index. Based on the two most recent social capital data, in both 2009 and 2014, which share the same component measures, other county-level data were added from the American Community Survey of the U.S. Census Bureau and the U.S. Department of Agriculture.
Data source location	There are three primary data sources: Northeast Regional Center for Rural Development at Penn State University, the U.S. Census Bureau's American Community Survey, and the Economic Research Service of the United States Department of Agriculture. All variables were separately downloaded and merged.
Data accessibility	Repository name: Mendeley Data
	Data identification number: 10.17632/ps8mtmtmvv.2
	Direct URL to data: https://data.mendeley.com/datasets/ps8mtmtmvv/2
Related research article	Mi-son Kim, Dongkyu Kim, and Natasha Altema McNeely, “Race, Inequality, and Social Capital in the U.S. Counties”
	https://doi.org/10.1080/03623319.2020.1799178

## Value of the Data

• Social scientists who are interested in the dynamics created by income inequality, racial diversity, and social capital in the U.S. Counties can easily utilize the dataset.
• This dataset also provides other county-level covariates that can be utilized by social science and humanities research.
• This dataset provides the most comprehensive measure of social capital of U.S. Counties for two time periods.

## 1 Data Description

This Data in Brief article is associated with the article “Race, Inequality, and Social Capital in the U.S. Counties.” [2] The data provided in this article were constructed to understand the variations of social capital across U.S. counties by examining the interaction between income inequality and ethnic diversity. Although the concept of social capital has been much debated, it can be largely defined as intangible social assets that individuals can utilize or enjoy by engaging with others. In that regard, Putnam [6] defines the concept as “networks, norms, and trust that enable participants to act together more effectively to pursue shared objectives.” Following Rupasingha et al. [7], the social capital index measures the extent to which individuals engage with others at the county-level.

**Fig. 1:**
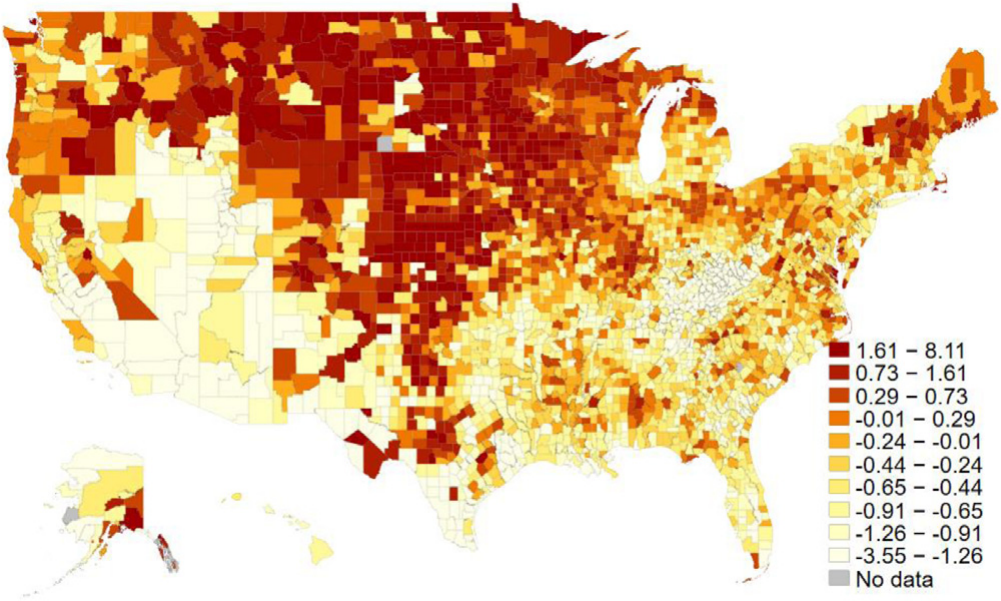
The Social Capital Index across U.S. counties. The average scores for both 2009 and 2014. The darker the region is, the more social capital there is in each county. The decile cut-off values differentiate colors.

The social capital index measures the common factor among four different types of variables: (1) the associational density of 10 different types of organizations (civic organizations, bowling centers, golf clubs, fitness centers, sports organizations, religious organizations, political organizations, labor unions, business organizations, and professional organizations), (2) the turnout rates for the previous presidential elections, (3) the response rate to the Census Bureau's decennial census, and (4) the number of non-profit organizations. The data are provided by the Northeast Regional Center for Rural Development at Penn State University. The index data has been updated four times since 1990. As the index has adopted a new associational typology for the 2000s data points, we only included data with a consistent typology. Thus, we have a social capital index for both 2009 and 2014, the two most recent data points. Table 1 reports all component measures’ summary statistics for each year, while Fig. 1 displays each county's average scores on the map.

**Table 1:** Social capital index components.

	2009					2014				
	N	mean	SD	min	max	N	mean	SD	min	max
Civic orgs	3106	9.0	21.7	0	538	3139	8.3	20.5	0	546
Bowling centers	3106	1.4	3.0	0	58	3139	1.2	2.6	0	48
Golf clubs	3106	3.8	7.3	0	142	3139	3.6	7.3	0	141
Fitness centers	3106	9.7	30.1	0	738	3139	10.1	33.6	0	845
Sport orgs	3106	0.3	1.1	0	29	3139	0.3	1.3	0	37
Religious orgs	3106	57.7	123.1	0	3258	3139	58.5	125.2	0	3275
Political orgs	3106	0.7	3.0	0	66	3139	0.8	3.8	0	76
Labor orgs	3106	4.8	15.0	0	292	3139	4.5	14.2	0	283
Business orgs	3106	5.3	14.4	0	323	3139	5.0	14.0	0	290
Professional orgs	3106	2.1	8.9	0	214	3139	2.1	9.0	0	210
Voter turnout	3106	0.6	0.1	0.17	2.079	3139	0.7	0.1	0.35	1.116
Census rate	3106	0.7	0.1	0	0.95	3139	0.7	0.1	0	0.95
NGOs	3104	489.1	1472.6	1	41,125	3139	458.4	1381.6	0	37,547

**Fig. 2:**
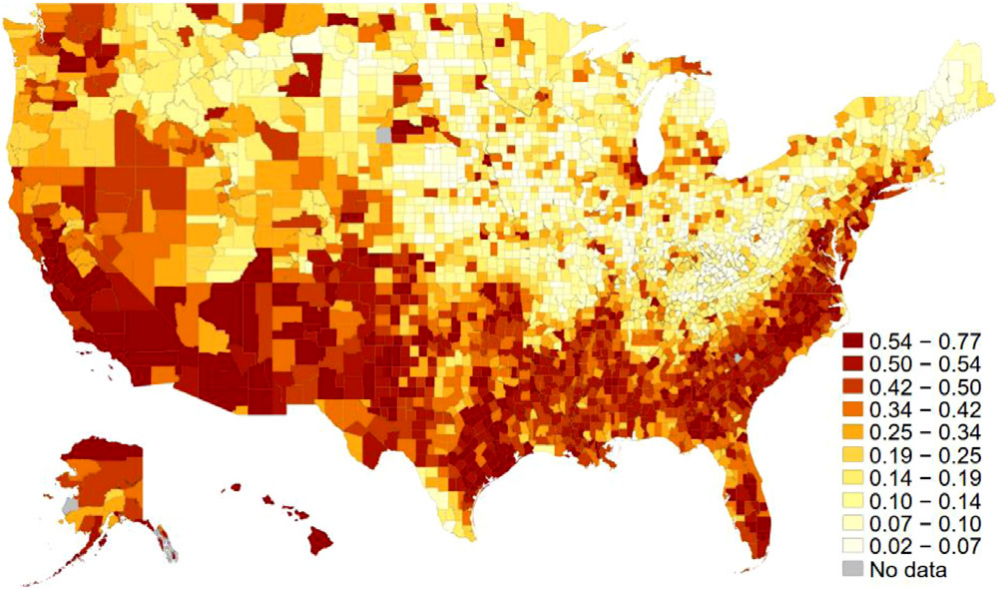
Racial diversity across U.S. counties. The average scores for both 2009 and 2014. The darker the region is, the more diversity there is in each county. The decile cut-off values differentiate colors.

**Fig. 3:**
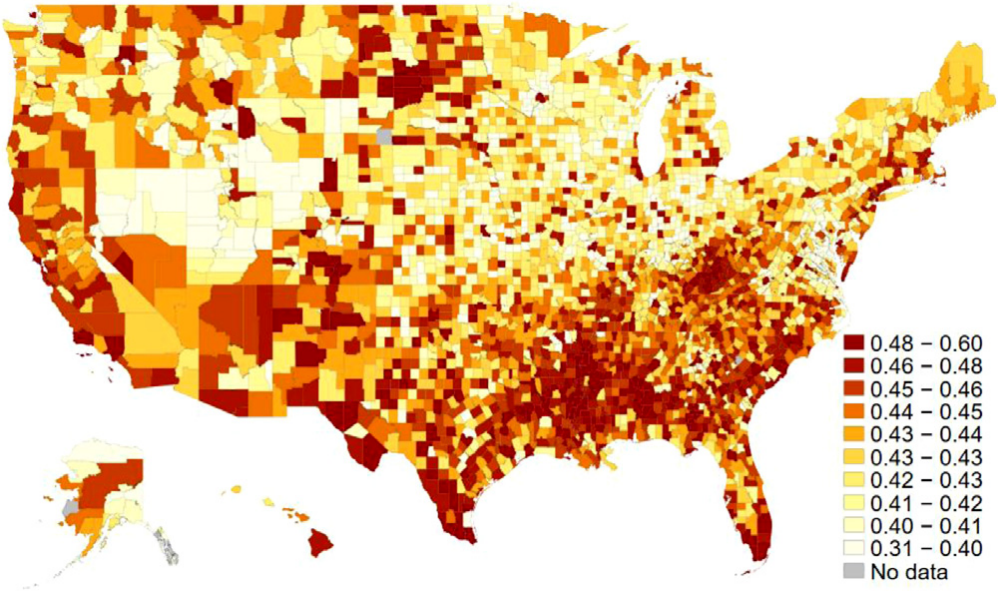
Income inequality across U.S. counties. The average scores for both 2009 and 2014. The darker the region is, the more inequality there is in each county. The decile cut-off values differentiate colors.

One of the independent variables is racial diversity. From data provided by the U.S. Census Bureau's American Community Survey (ACS), the racial diversity index was calculated as one minus the Herfindahl index of 7 ethnic groups (Non-Hispanic white, Hispanic, Black, Indian, Asian, Hawaiian, and two-more). It measures the probability that two people randomly chosen from a county belong to different ethnic groups (see, e.g., Alesina et al. 1999). Fig. 2 displays each county's average scores of diversity index on the map. Another key independent variable for the associated article is income inequality. Based on data also provided by the ACS, the variable measures the Gini index, which takes 0 for a perfectly equal distribution of income and 1 for perfectly unequal income distribution. Fig. 3 shows the geographical distribution of income inequality across the U.S. Counties. Table 2 shows the list of counties at both the top and the bottom ten ranks for these three key variables in 2014.

**Table 2:** 2014 Rankings of social capital, inequality, and diversity.

Social capital: top 10	Income Inequality: top 10	Racial Diversity: top 10
Hinsdale County, CO	Randolph County, GA	Aleutians West Census Area, AK
Lexington city, VA	Calhoun County, GA	Queens County, NY
Mineral County, CO	McMullen County, TX	Maui County, HI
Motley County, TX	New York County, NY	Alameda County, CA
Thomas County, NE	Borden County, TX	Aleutians East Borough, AK
Hooker County, NE	Baylor County, TX	Hawaii County, HI
Griggs County, ND	Orleans Parish, LA	Fort Bend County, TX
Grant County, NE	Corson County, SD	Kauai County, HI
Kiowa County, KS	Campbell County, SD	Solano County, CA
Smith County, KS	Eastland County, TX	Honolulu County, HI

Social Capital: bottom 10	Income Inequality: bottom 10	Racial Diversity: bottom 10

Sioux County, ND	Yakutat City and Borough, AK	Tyler County, WV
Jim Hogg County, TX	Bristol Bay Borough, AK	Jackson County, KY
Webb County, TX	Spencer County, KY	Holmes County, OH
Hancock County, TN	Emery County, UT	Magoffin County, KY
Zavala County, TX	Lake of the Woods County, MN	Dickenson County, VA
Loving County, TX	Sublette County, WY	Osage County, MO
Maverick County, TX	Chattahoochee County, GA	Lincoln County, WV
Starr County, TX	Grant County, NE	Leslie County, KY
Shannon County, SD	Power County, ID	Blaine County, NE
Chattahoochee County, GA	Clark County, ID	Keya Paha County, NE

**Fig. 4:**
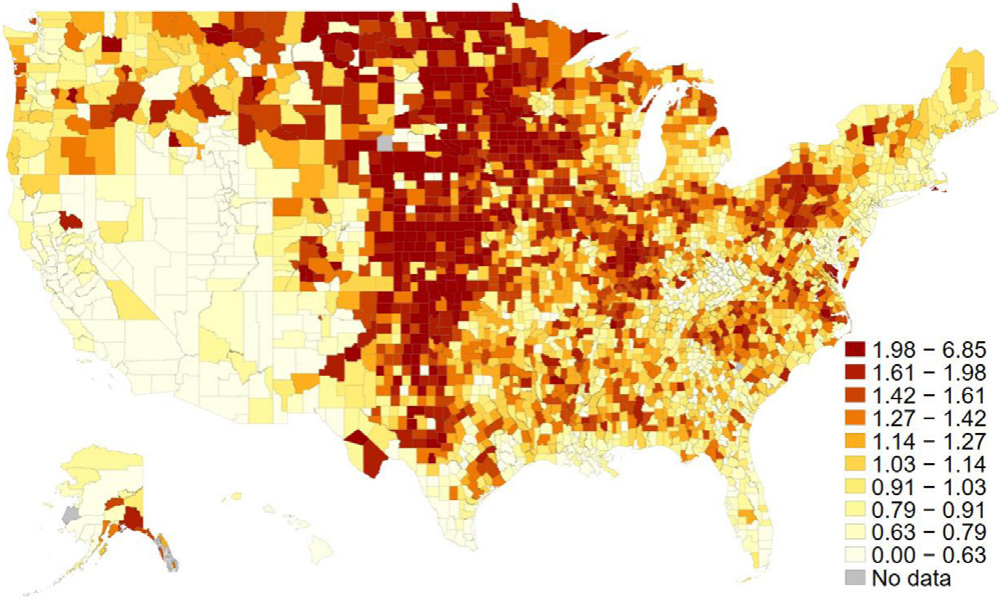
Putnam type (bridging) social capital index across U.S. counties. The average scores for both 2009 and 2014. The darker the region is, the more bridging social capital there is in each county. The decile cut-off values differentiate colors.

**Fig. 5:**
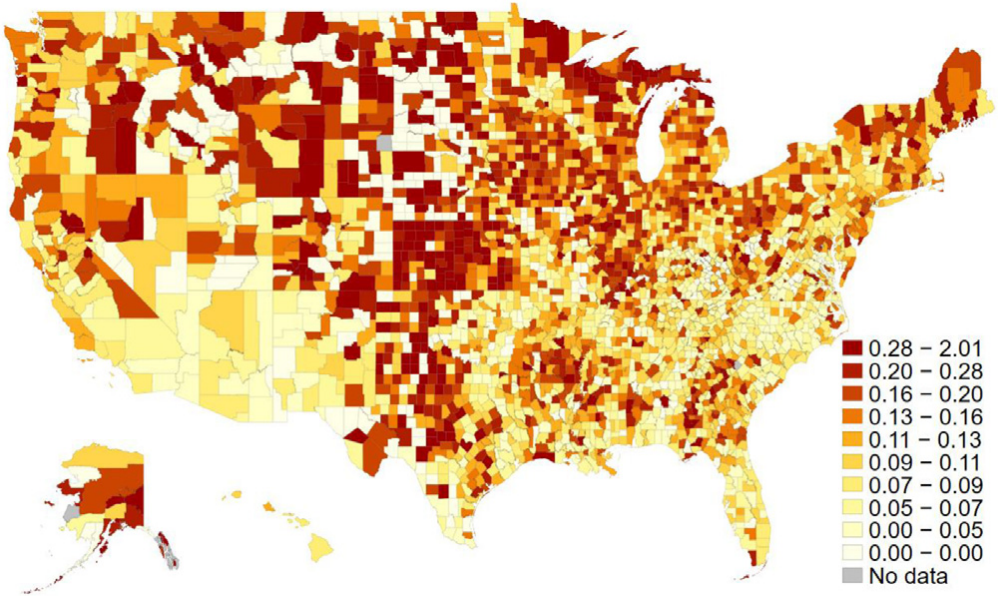
Olson type (bonding) social capital index across U.S. counties. The average scores for both 2009 and 2014. The darker the region is, the more boding social capital there is in each county. The decile cut-off values differentiate colors.

We further measured two different types of social capital by utilizing ten associational density variables. Scholars in the literature suggest that social capital has two different types: bridging and bonding social capital [3], [4], [5]. According to Putnam [5], bridging social capital can be defined as an open network that crosscuts, thus bridges, the existing social cleavages while bonding social capital is an inward-looking network that fortifies existing social interests. We labeled the former as ‘Putnam-type’ and the latter ‘Olson-type’ following Knack [3]. Based on Knack [3] and Rupasingha et al. [7], we measured bridging social capital (Putnam-type) with the associational density of the first six organizations (religious organizations, civic organizations, bowling centers, fitness centers, golf clubs, and sports organization) and bonding social capital (Olson-type) with the same density of the remaining associations (business organization, labor union, political organizations, and professional organizations). Both Fig. 4 and Fig. 5 display each variable on the map respectively.

We included other correlates of social capital in the dataset. Following the typology provided by the U.S. Department of Agriculture, the Rural-Urban Continuum Codes (RUCC), the urban and rural variables were dummy coded by taking suburban counties as a reference category. The RUCC scheme provides nine categories that distinguish metropolitan counties by population, and nonmetropolitan counties by population and adjacency to the metro area. We utilized three categories of metropolitan counties to construct a dummy variable for urban counties while using two categories of nonmetropolitan counties that are not adjacent to the metro area to construct the rural indicative variable. It is often believed that rural areas provide a favorable environment for social capital. In the statistical estimation of the associated article, the remaining category was considered as suburban areas and omitted in the regression analysis.

All other county-level variables are compiled by utilizing the ACS database. For the income variable, we used the mean income in the past 12 months with the inflation-adjusted dollars. Then, we transformed the average household income with the natural logarithm. The dataset also has the educational attainment variable that measures the percentage of residents who have at least some college education per county. It is well known that socioeconomic status is positively associated with social capital. Because social capital would be difficult to form in a fluid county, we include the share of the non-migratory population in our dataset. From the ACS's county-to-county migration flow data, we calculated the percentage of non-movers out of the county population. In a similar vein, it is expected that the family-oriented community would provide a good environment for social capital. Thus, the dataset includes the percentage of family households out of the total number of households for each county. Lastly, we include the size of the female workforce. The theoretical explanations about how traditional gender roles affect social capital are unsettled. Following Rupasingha et al. [7], we considered this variable to test the effect of women's traditional role as housewives empirically. Table 3 presents the summary statistics for all covariates over 3139 counties for both 2009 and 2014.

**Table 3:** Descriptive statistics.

Variable	N	mean	SD	Min	Max
Social capital index	6245	−0.007	1.250	−3.925	9.149
Racial diversity index	6245	0.286	0.183	0	0.769
Income Inequality index	6245	0.436	0.036	0.207	0.652
Urban	6244	0.372	0.483	0	1
Rural	6244	0.271	0.444	0	1
Ln(Average Household Income)	6245	10.942	0.224	10.259	11.934
Education (some college) ratio	6245	0.483	0.109	0.181	0.886
Non-migration population ratio	6234	0.859	0.046	0.478	0.997
Female workforce ratio	6245	0.701	0.076	0.361	1
Family household ratio	6245	0.523	0.068	0.233	0.902
Putnam (bridging associations)	6245	1.254	0.653	0	6.887
Olson (bonding associations)	6245	0.142	0.151	0	2.253

## 2 Experimental Design, Materials and Methods

Data construction for the associated article was constrained by the availability of data on social capital. Given social capital data for both 2009 and 2014, all relevant variables were compiled utilizing various data sources. Table 4 provides detailed information about all variables included in the dataset, including primary sources. These raw data are publicly available. However, putting them together to create correlates of social capital at the county-level requires careful handling of the data to align both temporal and geographical units. The Federal Information Processing Standards (FIPS), a four-digit county code, were used to match data points across different data sources. Furthermore, all data provided by the U.S. Census Bureau's ACS utilize the 5-year average estimates so that the dataset contains the least amount of missing values. With the constructed dataset, the associated article examined the variations of social capital at the county-level by utilizing two-stage multilevel regression analysis with year fixed effect [1,^8^,9]. Researchers could easily re-use or expand our dataset to better understand the variation of social capital at the county-level.

**Table 4:** Variable description and data sources.

Variable Description	Data source
FIPS - Federal Information Processing Standard, four-digit county codes	National Institute of Standards and Technology

sk - Social capital index – 13 components + population data	Northeast Regional Center for Rural Development
1. assn – Associational density of 10 types of organizations (per 1000 people)	
1. relig (# of religious organization), 2. civic (# of civic organization), 3. bus (# of business organization, 4. pol (# of political organization), 5. prof (# of professional organization), 6. labor (# of labor unions), 7. bowl (# of bowling centers), 8. fitns (# of fitness centers), 9. golf (# of golf clubs), 10. sport (# of sports organization), & 11. pop (County population)	
2. pvote – previous presidential election turnout	
3. respn – US Census response rate	
4. nccs – # of non-profit organizations	

gini – Gini coefficient	American Community Survey, US Census Bureau

eth_div – Ethnic diversity: 7-category diversity measure	American Community Survey, US Census Bureau
1. p_white (Non-Hispanic white%), 2. p_hispanic (Hispanic%), 3. p_black (Black%), 4. p_indian (American Indians%), 5. p_asian (Asian%), 6. p_hawaiian (Pacific Islander%), 7 p_tomore (Other%)	

urban & rural – Dummy variables for urban and rural counties	Economic Research Service, USDA

fam_household –% of family household	American Community Survey – US Census Bureau

female_wforce –% of female labor market participation	American Community Survey – US Census Bureau

educ –% of people with at least some college education	American Community Survey – US Census Bureau

income – Average household income	American Community Survey – US Census Bureau

Nonmover –% of non-migratory population	American Community Survey – US Census Bureau

Putnam – Bridging social capital: associational density for 6 components of sk: relig, civic, bowl, fitns, sport, & golf	Authors’ calculation

Olson – Bonding social capital: associational density for 4 components of sk: bus, pol, prof, & labor	Authors’ calculation

## Declaration of Competing Interest

The authors declare that they have no known competing financial interests or personal relationships which have, or could be perceived to have, influenced the work reported in this article.

## Appendix Supplementary materials

Image, application 1
